# ViralVar: A Web Tool for Multilevel Visualization of SARS-CoV-2 Genomes

**DOI:** 10.3390/v14122714

**Published:** 2022-12-05

**Authors:** Arghavan Alisoltani, Lukasz Jaroszewski, Adam Godzik, Arash Iranzadeh, Lacy M. Simons, Taylor J. Dean, Ramon Lorenzo-Redondo, Judd F. Hultquist, Egon A. Ozer

**Affiliations:** 1 Department of Microbiology-Immunology, Feinberg School of Medicine, Northwestern University, Chicago, IL 60611, USA; 2 Department of Medicine, Division of Infectious Diseases, Feinberg School of Medicine, Northwestern University, Chicago, IL 60611, USA; 3 Center for Pathogen Genomics and Microbial Evolution, Havey Institute for Global Health, Feinberg School of Medicine, Northwestern University, Chicago, IL 60611, USA; 4 Biosciences Division, School of Medicine, University of California Riverside, Riverside, CA 92507, USA; 5 Computational Biology Division, Department of Integrative Biomedical Sciences, University of Cape Town, Cape Town 7925, South Africa

**Keywords:** evolution, mutation, genomic surveillance, SARS-CoV-2, COVID-19, ViralVar, webtool

## Abstract

The unprecedented growth of publicly available SARS-CoV-2 genome sequence data has increased the demand for effective and accessible SARS-CoV-2 data analysis and visualization tools. The majority of the currently available tools either require computational expertise to deploy them or limit user input to preselected subsets of SARS-CoV-2 genomes. To address these limitations, we developed ViralVar, a publicly available, point-and-click webtool that gives users the freedom to investigate and visualize user-selected subsets of SARS-CoV-2 genomes obtained from the GISAID public database. ViralVar has two primary features that enable: (1) the visualization of the spatiotemporal dynamics of SARS-CoV-2 lineages and (2) a structural/functional analysis of genomic mutations. As proof-of-principle, ViralVar was used to explore the evolution of the SARS-CoV-2 pandemic in the USA in pediatric, adult, and elderly populations (n > 1.7 million genomes). Whereas the spatiotemporal dynamics of the variants did not differ between these age groups, several USA-specific sublineages arose relative to the rest of the world. Our development and utilization of ViralVar to provide insights on the evolution of SARS-CoV-2 in the USA demonstrates the importance of developing accessible tools to facilitate and accelerate the large-scale surveillance of circulating pathogens.

## 1. Introduction

Since the onset of the coronavirus disease 2019 (COVID-19) pandemic, the continued mutation and diversification of the severe acute respiratory syndrome coronavirus 2 (SARS-CoV-2) has resulted in the repeated emergence of new “variants of concern” (VOCs) with increased infectivity, transmissibility, and/or immune evasion properties [1,2,3,4,5]. Each VOC has been defined by a distinct set of protein mutations (missense or nonsynonymous substitutions, in-frame insertions, and deletions) that confer unique functional properties [1,6,7,8,9,10,11,12]. For example, the Alpha (B.1.1.7*) VOC was defined using a set of nine Spike mutations (N501Y, A570D, D614G, P681H, T716I, S982A, D1118H, 69-70Δ, 144Δ) that increased infectivity [13], transmissibility [14], and resistance to monoclonal antibody therapeutics [15]. Especially within the Spike open reading frame, a greater proportion of missense compared to synonymous mutations is indicative of strong positive selection for Spike proteins with altered structure and function [16,17]. Continued SARS-CoV-2 genomic surveillance is essential to identify new emergent variants with novel phenotypic properties that may alter best practices in public health and clinical care.

The remarkable global scientific response to the COVID-19 pandemic has led to the generation of vast amounts of publicly available SARS-CoV-2 whole-genome sequence data. Worldwide, most genome sequences are deposited in the GISAID public database (gisaid.org) [18], and more than 13 million viral sequences from around the world have been deposited as of 12 September 2022. This massive and ongoing SARS-CoV-2 sequencing effort has provided a unique opportunity to study the virus’s evolution in exquisite detail. However, at the same time, the volume and diversity of available sequences exacerbates the complexity of the data analysis and calls for effective tools to allow researchers with little or no computational expertise to perform detailed analyses of relevant genomic data.

In part to address this problem, several web-based tools have been developed to facilitate the study of SARS-CoV-2 spatiotemporal dynamics, mutational frequency, and/or three-dimensional (3D) protein structures [19,20,21,22]. Though useful for gaining broad insights, these applications are often limited to the analysis of predetermined datasets with minimal user control, such as COVIDCG [23], outbreak info [24], covariants [25], 2019nCoVR [26], CoV-GLUE [27], and COG-UK [28]. However, even tools that allow the processing of user-defined data often accept a limited number of sequencing data such as covdb (limit = 100) [29], coronApp [30] (limit ~100 MB or ~3500), and VirusViz (limit = 50) [31]. In addition to the lack of options for large-scale data analysis, these tools have limited analytical features for the multilevel analysis and visualization of SARS-CoV-2 lineages and their mutations (e.g., spatiotemporal visualization of lineages, linear or 3D visualizations of mutations in the context of proteins and genomes).

Other tools and databases have been developed to study SARS-CoV-2 protein structures. One of these applications is SARS-CoV-2 3D [32] which provides tools for 3D structure predictions and energy calculations to evaluate targets and design new potential therapeutics. CoV3D [33] is a repository for 3D protein structures of SARS-CoV-2 and host antibodies. Neither tool provides information on mutational changes in the context of the 3D structures. Other webservers such as the GISAID [18], covariants [25], and COG-UK [28] provide limited 3D structural visualizations for only fixed sets of mutations (mostly clade-defining) and only for the Spike protein. To the best of our knowledge, there are currently two webservers that enable the visualization of mutations in the context of 3D protein structures for all SARS-CoV-2 proteins: Coronavirus3D [19] and COVID-3D [21]. However, both servers have a fixed list of SARS-CoV-2 genomes/mutations in their databases and lack the option to visualize mutations based on user-provided genomes. Although COVID-3D [21] provides an option for the user to input variants, this is limited to only one protein at a time and thus requires multiple file uploads to visualize genomic mutations in the context of more than one protein. Moreover, both servers lack the capacity to examine mutational patterns at selected time points in the pandemic, in selected geographical regions, and/or among specific lineages.

Here, we present a new, web-based software application “ViralVar” that incorporates user-selected genome data to visualize and study lineages over time by depicting the distribution of mutations at both the nucleotide and protein levels as well as providing the context of variants in the 3D structure of SARS-CoV-2 proteins. Protein visualizations provide detailed information on the functional protein domains and predicted B-cell epitopes. Additionally, ViralVar provides a currently unique feature among similar applications that allows for the binomial testing of protein mutations to identify potential over- and under-mutated proteins, k-means clustering of genomes based on protein mutations to expedite large-scale surveillance of new mutations, and the visualization of changes in the mutational patterns of the virus over selected date ranges, within defined geographical regions, and/or within or among lineages. A practical demonstration of the application of ViralVar is given here by examining the relative dynamics of the evolution of SARS-CoV-2 in the USA, using a total of 1,739,797 sequenced genomes collected in the USA between January 2020 and May 2022. The ViralVar webserver is freely available at http://viralvar.org/.

## 2. Methods

### 2.1. General Software Workflow

**Figure 1:**
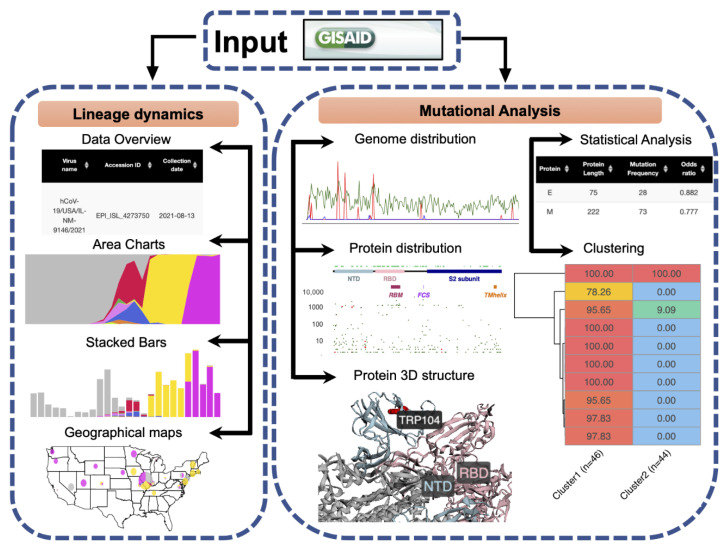
General workflow of ViralVar and its two main modules. Input data reflecting SARS-CoV-2 sequences of interest can be downloaded directly from the GISAID public repository. In the “Lineage Dynamics” module, the spatiotemporal dynamics of SARS-CoV-2 lineages and clades are represented in the form of stacked bars, area plots, and pie charts over user-selected timeframes and geographical areas. In the “Mutational Analysis” module, mutations are depicted in the context of the SARS-CoV-2 genome and relevant proteins (both primary sequence and 3D structural representations). This module also provides options to perform statistical analyses to identify over- and under mutated proteins over user-selected time periods and perform genome clustering within user- selected subsets. More details are available in the ViralVar User Manual.

The ViralVar webtool is implemented in the R programming language using Shiny, an open-source R package for developing interactive web applications. Shiny implements layout features available in Bootstrap, an HTML 4.01/shiny-css 1.7.1/shiny-javascript 1.7.1 framework. To add more advanced content to ViralVar, the user interface was customized with HTML and Shiny’s HTML tag attributes, as well as custom cascading style sheets (CSS) and other R packages listed in the context of the relevant sections below. Briefly, SARS-CoV-2 genomic data retrieved from the GISAID [18] were used as input for ViralVar. The webapp is divided into two modules (Figure 1). In the first module, “Lineage Dynamics”, data are processed to depict the spatiotemporal dynamics of SARS-CoV-2 lineages and clades in the form of stacked bars, area plots, and pie charts. The second module “Mutational Analysis” visualizes mutation distributions along the SARS-CoV-2 genome and proteins (linear and 3D) and generates statistical analyses to identify over- and under-mutated proteins. Users can interact with the server to explore and compare the temporal dynamics of the lineages and mutations between the different sets of genomes and/or VOCs. Each module provides various control options, allowing users to customize analyses and view and export figures according to their requirements. The output files of ViralVar are either high-resolution figures (PDF, PNG) or tables (tsv).

### 2.2. Data Input

The GISAID is one of the largest global resources for sharing SARS-CoV-2 genome sequences and associated clinical and demographic information [18]. GISAID data are accessible to users through free registration via the GISAID website (https://gisaid.org/) accessed on 31 May 2022. The database provides genome consensus sequences, reference-based multiple sequence genome alignments, and lists of mutations for each genome with the associated lineage or clade designations in tabular format. Data to be downloaded from the database can be readily filtered to focus on dates of collection, specific geographical regions, or selected lineages or clades. ViralVar accepts input data from the GISAID in a tabular format that includes three sets of information for each genome: (1) PANGO lineage (users can opt to manually add Nextclade designations), (2) sample collection date, and (3) a list of protein mutations (denoted as “AA Substitutions” in the GISAID data; required for “Mutational Analysis”). Detailed guidance on retrieving GISAID data in the proper format for input into ViralVar is provided on the “Home” tab of ViralVar. A limited set of 3892 SARS-CoV-2 sequences collected through the Northwestern Medicine Healthcare (NMH) system in Chicago, IL, between February 2020 and May 2022, are included in the ViralVar webtool for example purposes and can be viewed by checking the “Visualize Example Data” checkbox in each module. The GISAID IDs are provided in Table S1.

### 2.3. Lineage Dynamics

The “Lineage Dynamics” module of ViralVar serves to provide tools for visualizing changing trends in SARS-CoV-2 lineages/clades over time using temporal abundances and geographical distributions. ViralVar uses the R package ggplot2 [34] to generate visualizations reflecting the trend of changes in the absolute and relative abundances of SARS-CoV-2 lineages over time in the input data set. After data input, the data are displayed in a tabular format in the “Data Overview” tab. Note that for this module, only collection date and PANGO lineage information are required. The “Area Charts” and “Bar Charts” tabs illustrate the dynamics of lineage distributions over user-specified date ranges. The “Geographical map” tab shows lineage distributions overlaid as pie charts on user-selected geographical maps for the world, the USA, or individual USA states and territories again over a user-specified date range. Geographical maps are drawn using the R package maps and scatterpie. The phylogenetic nomenclature option allows users to customize output data to use PANGO lineage, Nextclade clade, or World Health Organization-defined VOC nomenclature. Tables and customizable figures are downloadable in a portable document format (PDF).

### 2.4. Mutational Analysis

The “Mutational Analysis” module of ViralVar provides users with a suite of tools to visualize the genomic and structural context of SARS-CoV-2 mutations. The R package ggplot2 [34] is used to generate and annotate density plots. After data input, the data are displayed in tabular format in the “Data Overview” tab. Note that for this module, collection date, PANGO lineage, and amino acid (AA) substitution information are required. The “Genome Distribution” tab depicts mutation density among uploaded sequences across the SARS-CoV-2 genome. Briefly, the number of distinct mutation events at each genomic position or protein residue is determined relative to a reference sequence (NCBI: NC_045512.2) [35] and reported over a sliding 100 nucleotide window. Position counts are calculated separately for insertions, deletions, and substitutions. This method does not consider virus counts in its calculation (i.e., the number of uploaded genomes with a particular mutation) such that each mutation event is counted only once. This avoids potential biases in reporting mutational frequency due to unequal amplification or sequencing across the genome as well as bias sampling [16]. In the “Protein Distribution” tab, the frequencies of genomes (virus counts) with mutations at specific protein residues are visualized using the R package ggplot2 [34] and plotly R package (interactive visualization). Separate plots can be generated for all SARS-CoV-2 proteins, both structural and nonstructural. Protein domain boundaries are indicated as described in the literature [16,17]. The IEDB server (Bepipred Linear Epitope Prediction 2.0 at http://www.iedb.org/) [36] (accessed on 31 October 2021) was used to predict B-cell epitopes, which are indicated above the protein schematic. In the “3D Protein Structure” tab, the R library package r3dmol is used to visualize mutations in the context of 3D protein structures. The 3D coordinates were obtained from the Protein Data Bank (PDB) with PDB accession numbers provided for each structure [37]. For proteins with no available 3D structure, models, as predicted by AlphaFold, were used when available [38]. Alternatively, the positions of transmembrane helices for proteins with no available 3D structures were identified using the TMHMM 2.0 algorithm [39]. Lists of the top mutations along with their frequencies for each protein can be downloaded in the form of tab delimited tables. The 3D protein illustrations can be downloaded as portable network graphics (PNG) files. Each of the above tabs includes a date slider to allow users to restrict data to a specific date range and a “Select VOC/VOI” option to limit output to a specified VOC or VOI.

The above mutational analysis tabs are further complemented by two tabs for statistical analysis and k-means clustering. In the “Statistical Analysis” tab, ViralVar utilizes the binomial test to identify individual proteins within the uploaded dataset that have significantly different mutation frequencies. The method has been previously applied to identify significantly under- and over-mutated SARS-CoV-2 proteins [16,17]. Briefly, the arguments for the binomial test are the observed number of distinct protein mutations in a certain protein (the “number of successes”), the total number of distinct protein mutations in all SARS-CoV-2 proteins (the “number of trials”), and the length of a given protein divided by the length of all SARS-CoV-2 proteins (the “expected probability of success”). An example of binomial calculations is provided below. For more details, please refer to [16].

To simplify the calculations in this method, we hypothesize that each protein mutation is an independent event and that all SARS-CoV-2 proteins and all residues have the same probability of being mutated. Therefore, this method applies the binomial test to assess the null hypothesis: that protein mutations are distributed randomly across all SARS-CoV-2 proteins.

$$P\left(MP,\;MT\right)=\left(\begin{array}{c} MT\\ MP \end{array}\right)P{\left(p\right)}^{MP}(1-{\left(P\left(p\right)\right)}^{MT-MP}$$

MT = the total number of protein mutations observed for all proteins (for example, 325 mutations in user input data)

MP = the number of protein mutations in the target protein (for example, 66 mutations in Spike in user input data)

*P*(*p*) = length protein/length proteome (e.g., length Spike/total length = 1273/9930 = ~0.13)

$$P\left(MP,\;MT\right)=\left(\begin{array}{c} 325\\ 66 \end{array}\right)0.13^{66}(1-{\left(0.13\right)}^{325-66}=0.00046$$

Based on the null hypothesis, we expect only 42 mutations in Spike, given that 66 out of the total 325 mutations identified in SARS-CoV-2 proteins are located in Spike, the length of the Spike protein is of 1273 amino acids, and the entire SARS-CoV-2 proteome is 9930 long. However, the binomial test *p*-value (0.00046) suggests rejection of the null hypothesis and indicates a significantly higher number of mutations in the Spike proteins compared to the background (entire proteome). ViralVar conducts the above calculation for user input data; therefore, MP, MT, and *P*(*p*) will be different for each input dataset. An option to exclude clade signature mutations is provided to avoid bias in the binomial test across highly divergent clades. ViralVar also provides control options to customize binomial test parameters, including the option to adjust the *p*-values for multiple comparisons. As above, the tab includes a date slider to allow users to restrict data to a specific date range and a “Select VOC/VOI” option to limit output to a specified VOC or VOI. A results table of the analysis can be downloaded as a tsv file.

In the “Genome Clustering” tab, ViralVar employs k-means clustering to facilitate rapid investigation of emerging clusters of genomes with specific protein mutation. As the selection of mutations in SARS-CoV-2 evolution has been shown to be largely impacted by positive selection, driven by changes in SARS-CoV-2 protein structures and functions [16,17], targeting protein mutations could cluster genomes relative to the phenotype. For instance, a common feature of SARS-CoV-2 genomes with the N501Y spike mutation (e.g., Alpha, Beta and Gamma strains) was enhanced infectivity and transmissibility over the previous variants [14].

The clustering of genomes based on pairwise distance-based methods is computationally intensive and might take days to run depending on the computational resources. The runtime for the first step of these approaches (the calculation of distance matrices for all pairs of genomes) increases exponentially with the increase in the number of genomes (Figure S3). In contrast, k-means clustering of SARS-CoV-2 genomes has been proposed in the recent literature as a rapid method to investigate emerging variants and tackle the computational challenges in large-data analysis [40,41]. Due to its simplicity and being computationally inexpensive, the k-means clustering of genomes, based on mutations in specific proteins, can be quickly and repeatedly run on large-scale genomic datasets (such as ~11.1 M SARS-CoV-2 genomes).

ViralVar uses k-means to group genomes-based on protein mutations. To avoid the effects of spurious mutations (e.g., due to sequencing or assembly errors), the clustering of the genomes is calculated only from protein mutations with a default minimum mutation frequency (MMF) of >0.005, although this cutoff is user-adjustable. To determine the optimal number of clusters, ViralVar repeats k-means clustering for numbers of clusters (determined based on the number of variables in the input file) and calculates the average silhouette width (ASW) index using the R package NbClust [42]. In the calculation of the ASW, ViralVar uses unique genomes (duplicated genomes with identical mutational patterns are removed) to make calculations less computationally expensive. However, the final clustering is applied to all of the genomes in the input data to produce counts of the genomes in each cluster. As with the previously described functions, VOC/VOI and date range are selectable. The protein selection option allows for targeting mutations along a protein of interest. Tables and customizable figures in a PDF format are downloadable.

### 2.5. Applying ViralVar to Assess Dynamics of SARS-CoV-2 Evolution

The following was retrieved from the GISAID for the collection dates between 1 January 2020 and 15 May 2022 (downloaded 31 May 2022): a total of 1,739,797 SARS-CoV-2 high-quality complete genome assemblies (the GISAID criteria, including N content <5%) from the USA for which patient age, collection date (day/month/year), and geographic location available. To study the dynamics of the SARS-CoV-2 evolution using ViralVar, sequenced samples were classified into three populations by age: children (0–18 years), adults (18–64 years), and elderly (65+ years) (Table 1). The list of the GISAID identifiers that compose each group is provided in Table S1. Sequence data for each age group were uploaded separately and analyzed using ViralVar. Mutation distributions were also compared between the SARS-CoV-2 genomes collected and sequenced for different age groups in the USA. Additionally, to show the application of ViralVar to smaller sample sets (i.e., from regions with lower rates of genome sequencing relative to the USA), we targeted 248 SARS-CoV-2 genome sequences collected in Nigeria between 15 December 2020 and 15 January 2021 and 90 sequences collected from Cape Town, South Africa between 15 December 2020 and 15 January 2021. The list of the GISAID identifiers is provided in Table S1. Each of the Cape Town and Nigeria samples were processed using ViralVar.

## 3. Results and Discussion

### 3.1. Spatiotemporal Dynamics of SARS-CoV-2 VOCs in the USA

The United States has experienced one of the world’s highest COVID-19 burdens during the pandemic, with a total of 86.4 M confirmed cases and 1.01 M deaths as of 31 May 2022. Whereas some reports detailing the evolution of the COVID-19 pandemic in select cities and states are available [43,44], there are few comprehensive reports at a national level. To demonstrate the capabilities of ViralVar, we downloaded all high-quality whole genome sequence data available in the GISAID on specimens collected in the USA between 1 January 2020 and 15 May 2022 (n = 1,739,797 SARS-CoV-2 sequences total). These data were sorted by age (children, adults, elderly) and uploaded into the ViralVar webtool for analysis.

**Figure 2:**
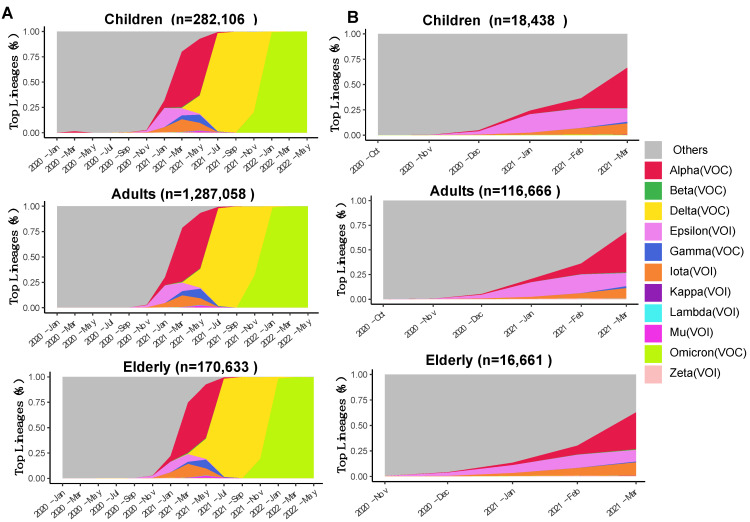
Area plots reflecting (different colors represent variants of concern see legend for details) the relative abundance of variants of concern and variants of interest collected in the USA over time. (**A**) Frequency of indicated VOCs and VOIs over time in specimens collected between January 2020 and May 2022 in the USA (n = 1,739,797 sequences from GISAID as of 31 May 2022). (**B**) Frequency of indicated VOCs and VOIs over time in specimens collected between November 2020 and March 2021. Specimens were divided into three age groups: children (up to 18 years), adults (18–64 years), and the elderly (65 years or more). The number of sequences per age group is indicated above each plot. Each subset of genomes was processed separately using the ViralVar “Lineage Dynamics” module.

The temporal dynamics of the VOCs across age groups in the USA were visualized using the “Area Chart” tab in the “Lineage Dynamics” module of ViralVar (Figure 2A). The results indicate that the dynamics of the VOCs were relatively similar for all age groups. The SARS-CoV-2 lineage B.1.1.7, designated by the WHO as “Alpha”, was the first named VOC and likely emerged in the United Kingdom (UK) in September 2020. Alpha rapidly displaced other circulating lineages in the USA and became one of the top circulating VOCs in the world in early 2021 [45]. The emergence of Alpha in the USA can be tracked back to November 2020 (Figure 2B), coincident with a spike of new cases and deaths between November 2020 and March 2021 (Figure S1). Using the date range feature to focus on dynamics during these months, Alpha emerges as the dominant variant at the tail-end of the surge in cases, suggesting that it was not responsible for the rise in cases but rather took over after the contraction in cases of the previous variant (Figure 2B).

**Figure 3:**
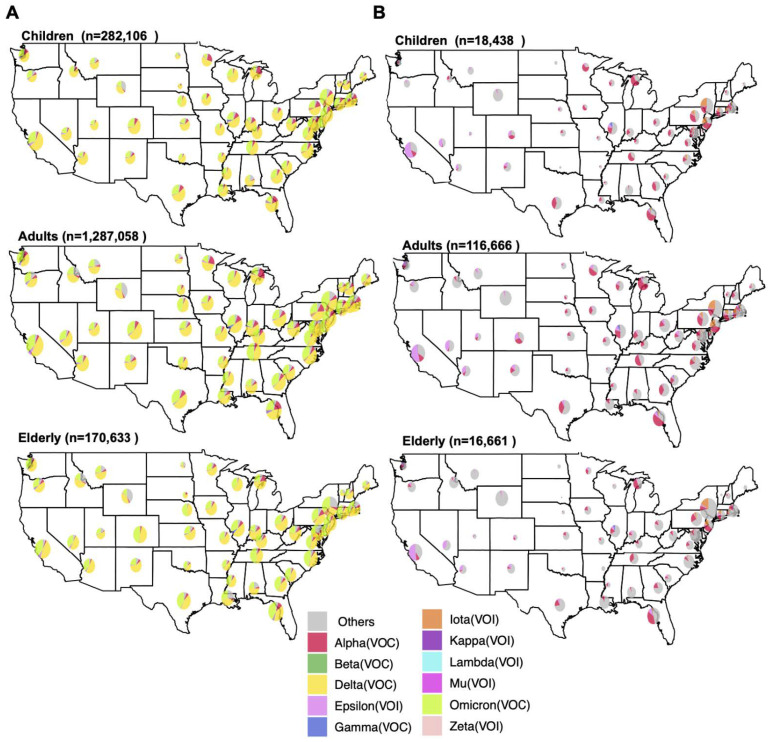
Distribution of SARS-CoV-2 variants of concern and interest by US state. Pie charts represent the proportion of SARS-CoV-2 VOIs and VOCs in each US state as reported to GISAID (as of 31 May 2022) (**A**) between January 2020 and May 2022 and (**B**) between November 2020 and March 2021. Specimens were divided into three age groups: children (up to 18 years), adults (18–64 years), and the elderly (65 years or more). The number of sequences per age group is indicated above each plot. The size of pie charts represents the relative frequency of sequenced data in each state. Each subset of genomes was visualized separately using the ViralVar “Geographical Map” feature.

Utilizing the “Geographical Map” feature in the “Lineage Dynamics” module, the distribution of VOCs collected between January 2020 and May 2022 was visualized for each age group by state (Figure 3A). The lineage distributions were similar across states between all age groups, with the Omicron and Delta VOCs making up the majority of cases, followed by Alpha (Figure 3A and Figure S2). In narrower timeframes, however, distinct spatiotemporal trends become more obvious. Using the date control feature, we adjusted this analysis to examine cases between November 2020 and March 2021 (Figure 3B). Whereas Alpha lineages made up the majority of cases in most states over this time period, region-specific trends emerged. For example, the Epsilon VOI was responsible for a substantial number of cases in southwestern states, whereas the Iota VOI was more prevalent in the northeast. Illinois specifically reported a substantial number of cases of the Gamma VOC that were not reflected in the neighboring states. These region-specific trends were consistent across age groups (Figure 3B).

### 3.2. Mutational Analysis of Alpha Variant Sublineages in the USA

**Figure 4:**
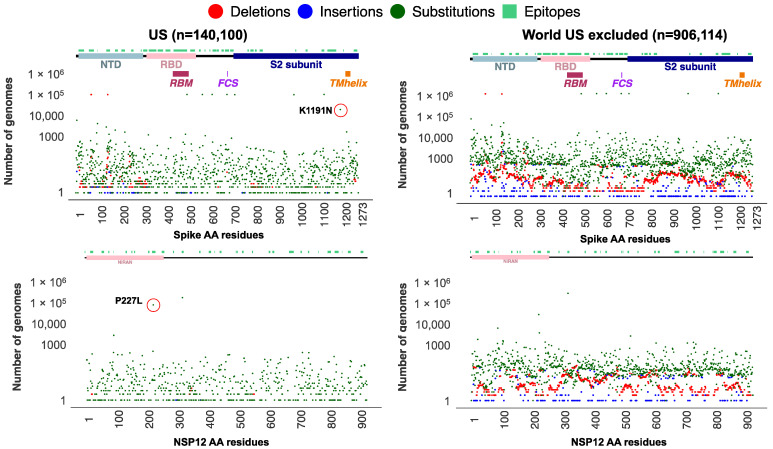
Absolute frequency of mutations in SARS-CoV-2 Spike and NSP12 among Alpha VOCs. SARS-CoV-2 genome data for all sequences assigned to an Alpha variant lineage (B.1.1.7 and Q.*) from the USA (n = 140,100) and rest of the world (n = 906,114, the USA cases excluded) were retrieved from GISAID as of 31 May 2022. Plots represent the absolute frequency of mutations at each amino acid position across Spike (**top**) and NSP12 (**bottom**) in sequences from the USA (**left**) and rest of the world (**right**). Deletions (red), insertions (blue), and substitutions (green) are plotted in different colors at each position. Boundaries for protein domains of Spike and NSP12 proteins were obtained from [16,17]. Predicted B-cell epitopes are highlighted above in teal, as predicted by [36]. Each subset of genomes was visualized separately using the ViralVar “Protein Distribution” feature.

**Figure 5:**
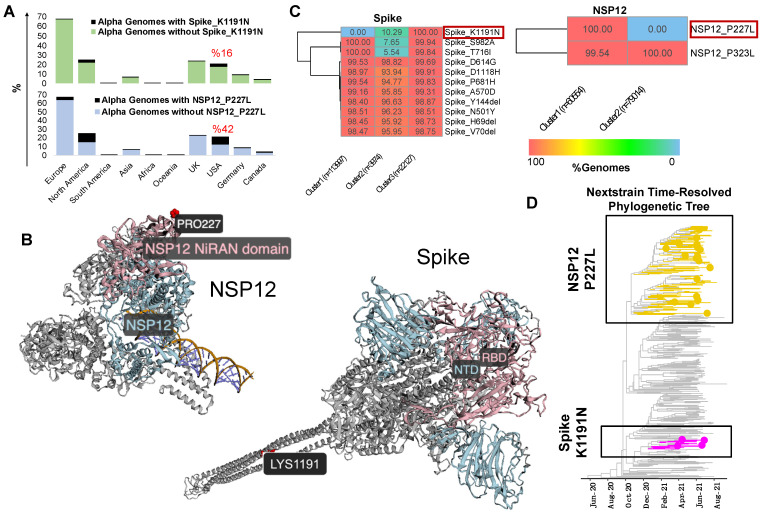
Analysis of Alpha VOC mutations predominantly found in USA specimens. (**A**) Relative frequency of Alpha VOC SARS-CoV-2 genomes harboring Spike K1191N (**top**) or NSP12 P227L (**bottom**) mutations. Calculations are based on GISAID data as of 31 May 2022. (**B**) Spike K1191N (**top**) or NSP12 P227L (**bottom**) mutations highlighted on available protein structures using the ViralVar “3D Protein Structure” feature. The Spike receptor-binding domain (RBD) and N-terminal domain (NTD) are colored in light blue and pink, respectively (D-I-TASSER model). NSP12 is colored in light blue with the NiRAN domain highlighted in pink (PDB: 6XEZ). (**C**) Euclidean distance-based k-means clustering of Alpha VOC SARS-CoV-2 genomes based on Spike and NSP12 mutations was performed using the “Genome Clustering” feature. Heatmaps represent the percent of genomes with a specific mutation within each cluster. Only protein mutations present in more than two thirds (70%) of genomes are shown here. (**D**) Time-resolved phylogenetic tree built by Nextstrain (https://nextstrain.org/ncov/gisaid/north-america/) accessed 14 October 2021 using a North America-focused subsampling between December 2020 and August 2021 (n = 399 sequences) visualized using R package ggtree. Yellow and hot pink branches and tips highlight genomes containing the Spike K1191N and NSP12 P227L mutations, respectively.

We subset the USA data explained earlier (n = 1,739,797 SARS-CoV-2 sequences) to only include genomes assigned to the Alpha lineage (n = 140,100). Additionally, genomes assigned to the Alpha lineage collected from the entire world (n = 906,114 excluding the USA) were retrieved from the GISAID as of 31 May 2022. Using the ViralVar “Mutational Analysis” module, the mutation profile for the Alpha VOC in the USA was compared to specimens from other countries. All ages were grouped together for this analysis due to the relatively small sample size of the under 18 and over 65 populations compared to adults in this dataset. Using the “Protein Distribution” tab, we visualized the mutational frequency in the Alpha VOC sequences at sites across Spike and NSP12 in both the USA and in the rest of the world (Figure 4). Whereas the defining mutations of the Alpha VOC were universally present, a distinct subset of mutations were more prevalent in the USA, specifically the Spike mutation K1191N and the NSP12 mutation P227L. To further investigate these mutations, all genomes containing Spike K1191N (n = 51,713) and NSP12 P227L (n = 190,869) mutations were retrieved from the GISAID and uploaded into ViralVar. A majority of genomes with the Spike K1191N mutation were in the Alpha variant genomes (80.4%, 41,558 of 51,713 genomes), of which the vast majority came from the USA (93.5%, 38,837 of 41,558 genomes) (Figure 5A, top). Similarly, 169,314 genomes with the NSP12 P227L mutation were classified as Alpha variants (88.7%), of which 104,435 genomes were collected in the USA (61.6%) (Figure 5A, bottom).

The Spike K1191N and NSP12 P227L appear to be recurrent mutations that have emerged in several other VOCs (i.e., Delta, Omicron, and Gamma); however, there is a lack of evidence regarding their role in virus infectivity, transmissibility, and/or clinical outcomes. To gain insight into their possible functional roles, we examined the protein context of each mutation using the “3D Protein Structure” feature in ViralVar (Figure 5B). The NSP12 P227L mutation is located in the Nidovirus RdRp-associated nucleotidyl transferase (NiRAN) domain. Although it is surface-exposed, it is far from the RNA binding or enzymatic active site. That being said, a nearby mutation in the NiRAN domain, N198S, has been recently reported as a potential antiviral resistance mutation to the NSP12-targeting drug, remdesivir [46]. Given the high level of conservation among coronavirus RNA-dependent RNA polymerases (RdRps) [47] and the recurring, but infrequent, prevalence of this mutation, it may also be that the P227L mutation confers some selective benefit but at a fitness cost to the virus [46]. The Spike mutation K1191N is located in the S2 subunit in the heptad repeat 2 (HR2) subdomain of the Spike protein, which is involved in host cell membrane fusion and viral entry (Figure 5B) [48]. Other Spike protein mutations in the HR2 subdomain, such as V1176F, have been shown to augment the stability of Spike and have been associated with increased disease severity and mortality [49,50,51]. More studies are required to determine the functional consequences of both Spike K1191N and NSP12 P227L.

### 3.3. ViralVar K-Means Clustering Feature Identifies Subclusters of the Alpha Variant in the USA

To better understand the genomic context of these mutations, we used the “Clustering Analysis” feature in ViralVar to identify co-occurring groups of mutations. K-means clustering based on Euclidean distance was applied to all of the Alpha VOC sequences collected in the USA, using a minimum mutation frequency cutoff of 0.005 and with a focus on the Spike and NSP12 proteins. The clustering of the Alpha genomes based on Spike mutations resulted in three distinct clusters (Figure 5C), two of which were defined by the presence (cluster 3) or absence (cluster 1) of K1191N. A third cluster showed a minor presence of K1191N but concurrently lacked the S982A and/or T716I mutations (cluster 2). The clustering of the Alpha genomes based on NSP12 mutations identified two distinct clusters distinguished solely by the P227L mutation (Figure 5C). To determine if these clusters can also be identified using phylogenetic analysis, we examined these mutations using the Nextstrain webserver (Figure 5D). The time-resolved phylogenetic trees from Nextstrain suggest that the Spike K1191N mutation is monophyletic, whereas the P227L mutation arose in at least two distinct branches (Figure 5D). The k-means clustering is largely in accordance with the phylogenetic analysis but suggests that additional mutational information, including synonymous mutations and those that occur outside of the open reading frame of interest, capture additional information not accounted for in this approach.

One of the limitations of the phylogenetic tree-based analysis, clustering, and visualization of SARS-CoV-2 genomes and investigating protein mutations is the computational cost that multiplies with the number of available genomes. The majority of studies using phylogenetic trees to study SARS-CoV-2 variants of concern (VOCs), therefore, must rely on subsampling approaches [52,53]. The k-means-based clustering of SARS-CoV-2 genomes based on Euclidean distance is one way to overcome this challenge as the method calculates the distance of each datapoint to the centroid using pairwise distances instead, decreasing the computational cost of analyzing the additional sequences (Figure S3). Furthermore, the k-means clustering of genomes based on protein mutations can be leveraged to the group genomes in a way directly related to the phenotype [54,55]. The congruence between the approach taken by ViralVar (Figure 5C) and the phylogenetic analysis results (Figure 5D) support the potential use of k-means clustering for the rapid analysis of large genomic datasets to facilitate tracking emerging protein mutations using a generic clustering method. This method could also be readily adapted and applied to other viruses. That being said, this approach is not suitable for making specific evolutionary inferences and so can be considered complementary to traditional phylogenetic-tree-based methods and useful for initial analyses and hypothesis generation.

### 3.4. Significant Nonrandom Distribution of Mutations in SARS-CoV-2 Proteins

**Figure 6:**
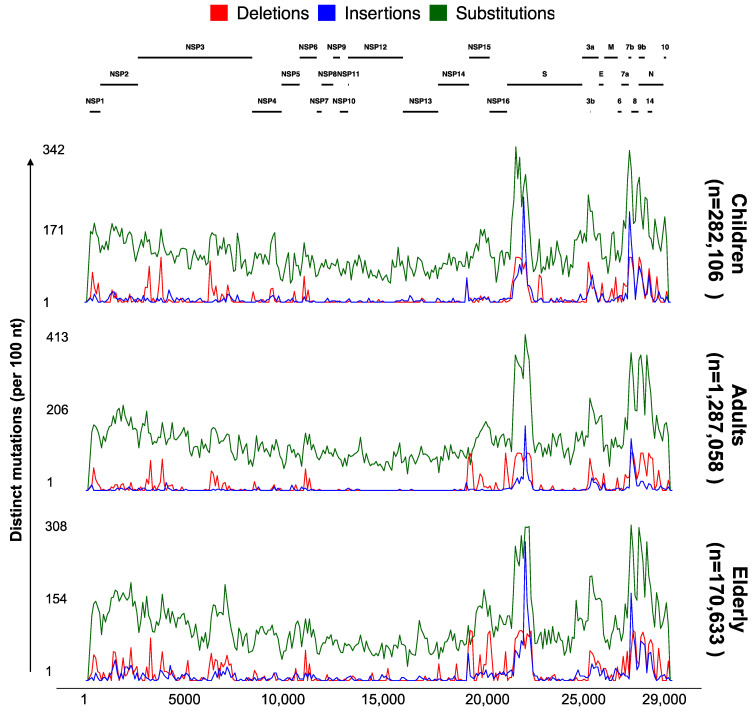
Genomic distribution of SARS-CoV-2 mutations for three age groups. Each plot depicts the number of distinct protein mutations in a 100 nucleotide sliding window across the SARS-CoV-2 genome in specimens collected between January 2020 and May 2022 in the USA (n = 1,739,797 sequences from GISAID as of 31 May 2022). Sequences were divided into six groups based on the age of patients (children (up to 18 years), adults (18–64 years), and elderly (65 years or more)). The total number of sequences used per age group is indicated. Each subset of genomes was processed separately using the ViralVar “Lineage Dynamics” module.

To explore the different mutational profiles in genomes collected from different age groups in the USA, we used the “Genome Distribution” feature of ViralVar to visualize the mutations in all of the collected specimens from the USA split by age group (Figure 6). Overall, the analyses of the mutation profiles of the SARS-CoV-2 genomes were relatively similar for the three age groups in the USA samples (Figure 6). Compared to structural and accessory proteins, nonstructural proteins seem to undergo a higher mutational constraint (Figure 6 and Table S2), consistent with the previous reports [16]. The slight variability in the mutational patterns between different data subsets could be partly attributed to the differences in the population size and sampling dates between regions and age groups.

One of the most noteworthy differences when comparing the results from the first year of the pandemic [16] and results obtained in this study is the increased frequency of protein indel events, especially the accumulation of insertions in the Spike NTD. This trend was consistent for samples collected across all age groups, though distinct deletion events appeared more prevalent in elderly populations (for example, in the NSP15 open reading frame, Figure 6). The increased frequency of recurrent indels and their nonrandom distribution is believed to be an adaptive response mechanism to elevated global herd immunity, resulting from vaccination, infection, or both [17,56,57]. Spike NTD indels could alter neutralizing epitopes in the region and are thought to result in reduced antibody protection against VOCs that harbor these indels [56].

Using the “Statistical Analysis” feature of ViralVar, we further identified significant accumulations of mutations in mostly the structural proteins of SARS-CoV-2 with two exceptions for the nonstructural proteins (NSP1 and NSP2). Of note, a higher concentration of mutations was observed in NSP1 (average odds ratio = 1.46, q-value = 0 across all age groups), NSP2 (average odds ratio = 1.3, q-value = 0 across all age groups), N (average odds ratio = 1.6, q-value = 0 across all age groups), NS6 (average odds ratio = 1.6, q-value = 0 across all age groups), NS7a (average odds ratio = 3.1, q-value = 0 across all age groups), NS7b (average odds ratio = 1.8, q-value = 0 across all age groups), NS8 (average odds ratio = 3.1, q-value = 0 across all age groups), and Spike (average odds ratio = 1.4, q-value = 0 across all age groups) (Table S2). All these proteins are involved in interactions with the host immune system [58,59,60]. Recurrent NSP1 substitutions and indels have been found to accumulate on the protein surface and near epitope regions [17] and are thought to adversely affect the host’s immune response and vaccine efficiency [61,62]. For instance, NSP1 Δ79-89 induces a lower IFN-I response in the infected Calu-3 cells [62], highlighting the biological importance of mutations in NSP1 and other nonstructural proteins. The significantly higher concentration of mutations in the specific proteins involved in host immune interactions, the emergence of new types of protein mutations (in-frame indels), and the expansion of mutations to new proteins or protein regions suggest the virus is evolving to combat the host immune system. Taken together, nonrandom distribution of the mutations in different SARS-CoV-2 proteins suggests proteins undergo different evolutionary pressures driven partly by the host immune system.

### 3.5. ViralVar Potential in Identifying Novel Variants in Small and Local Cohorts

Using ViralVar, we explored the evolution of 90 SARS-CoV-2 genome sequences collected in Cape Town, South Africa between 1 October 2020 and 30 November 2020 and 248 sequences collected in Nigeria between 15 December 2020 and 15 January 2021. Our analysis suggests the presence of two clusters based on the Spike protein mutations in the Cape Town samples (Figure S4A). Cluster 1 samples were all assigned to the Beta VOC (B.1.351) (Figure S4B) which was first described in South Africa [1]. We identified three distinct clusters of genomes based on Spike mutations in the Nigeria sample cohort (Figure S4C). The second cluster in this analysis corresponds to the Eta variant (B.1.525) (Figure S4D) which was identified as a variant local to West Africa [63]. These analyses show the potential of ViralVar for the analysis and tracking of mutations in small and regionally collected datasets.

## 4. Conclusions

The emergence of new variants of SARS-CoV-2 with higher transmissibility and enhanced immune evasion highlights the need for ongoing SARS-CoV-2 genomic surveillance. This work has been greatly facilitated by public sequence repositories such as the GISAID which contained available data for more than 11.1 M genome sequences as of 31 May 2022. At the same time, this vast amount of genomic data has increased the demand for more flexible and multilevel analysis platforms to help study the virus evolution. To complement and expand upon previously developed analysis tools, we created ViralVar, a webtool for visualizing and researching SARS-CoV-2 lineages and mutational patterns over time. We have shown that ViralVar can be deployed as a point-and-click tool to rapidly investigate the spatiotemporal evolution of large numbers of SARS-CoV-2 genomes. Overall, our findings utilizing ViralVar offer important insights into pathogen evolution dynamics and spread in the USA. This study demonstrates that ViralVar can be successfully used to study the evolution of SARS-CoV-2 and help in improving global COVID-19 mitigation plans as the pandemic continues to evolve.

As part of a larger project for facilitating the study of virus evolution and mutational patterns, the development of ViralVar will continue for the study of other viruses. Additional future work includes the addition of multiple data input options (i.e., consensus sequences or multiple sequence alignments) to facilitate users in analyzing their own data. The continued enrichment of the list of the structural and functional properties of SARS-CoV-2 and other viral proteins in ViralVar will also take place on a regular basis. The ViralVar databases will be updated at regular intervals based upon information provided for other viruses and updates in public databases for protein structural and functional properties. ViralVar complements current tools for studying the massive number of SARS-CoV-2 genomes and can provide a user-friendly platform for the multilevel study of SARS-CoV-2 evolution.

## Figures and Tables

**Table 1:** Details of SARS-CoV-2 data used in this study. Data retrieved from GISAID and each of the three data subsets were separately analyzed using ViralVar.

	Sequences	Mean Age	Median Age
Children (<18)	282,106	10.22	10.5
Adults (18–65)	1,287,058	38.92	37.5
Elderly (>65)	170,633	74.42	72.5

## Data Availability

The ViralVar webserver is freely accessible through http://viralvar.org/.

## Supplementary Materials

The following supporting information can be downloaded at: https://www.mdpi.com/article/10.3390/v14122714/s1, Figure S1: Frequency of new cases and deaths in the USA; Figure S2: Area plots reflecting the absolute abundance of variants of concern and variants of interest collected in the USA over time; Figure S3: Runtime performance for K-means clustering. Figure S4: ViralVar Potential in Identifying Novel Variants in Small and Local Cohorts; Table S1: List of GISAID SARS-CoV-2 genome sequence IDs used in this paper; Table S2. Significant over and under-mutated SARS-CoV-2 proteins.
