# Supplementary material for: ViralVar: A Web Tool for Multilevel Visualization of SARS-CoV-2 Genomes
Source: Viruses. 2022 Dec 5;14(12):2714. doi: 10.3390/v14122714 (PMC9781208; doi:10.3390/v14122714)
Supplement: Supplementary file 1 [file viruses-14-02714-s001.zip › Supplementary_Figures_Final_revised.pdf]

Supporting Information for

ViralVar: A Web Tool for Multilevel Visualization of SARS-CoV-2 Genomes

Arghavan Alisoltani[1,2,3]*, Lukasz Jaroszewski[4], Adam Godzik[4], Arash Iranzadeh[5], Lacy M. Simons[2,3], Taylor Dean[2,3], Ramon Lorenzo-Redondo[2,3], Judd F. Hultquist[2,3], Egon A. Ozer[2,3*]

Affiliations

[1]Department of Microbiology-Immunology, Northwestern University Feinberg School of Medicine, Chicago, Illinois, USA

[2]Department of Medicine, Division of Infectious Diseases, Northwestern University Feinberg School of Medicine, Chicago, Illinois, USA

[3]Center for Pathogen Genomics and Microbial Evolution, Havey Institute for Global Health, Northwestern University Feinberg School of Medicine, Chicago, Illinois, USA

[4]University of California Riverside School of Medicine, Biosciences Division, Riverside, California, USA

[5]Department of Integrative Biomedical Sciences, Computational Biology Division, University of Cape Town, Cape Town, South Africa

*Corresponding author contact: Egon Ozer (e-ozer@northwestern.edu) and Arghavan Alisoltani (arghavan.alisoltanidehkordi@northwestern.edu)

Supplementary Figures

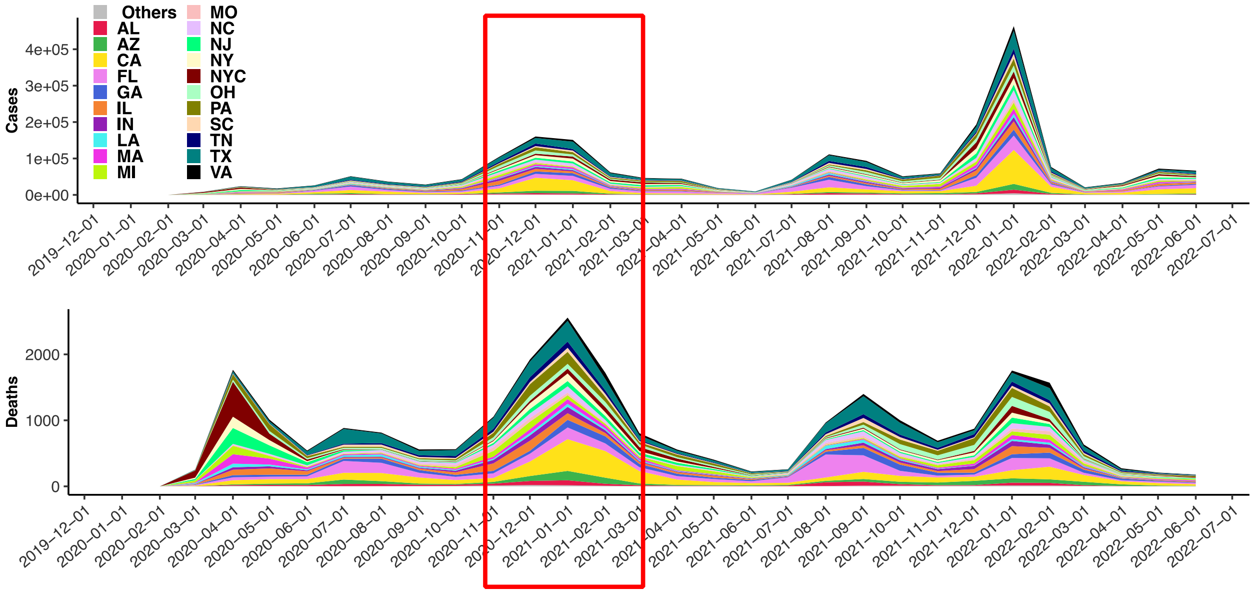

Figure S1 | Frequency of new cases and deaths in the USA. The data underlying the figures obtained from https://data.cdc.gov/.

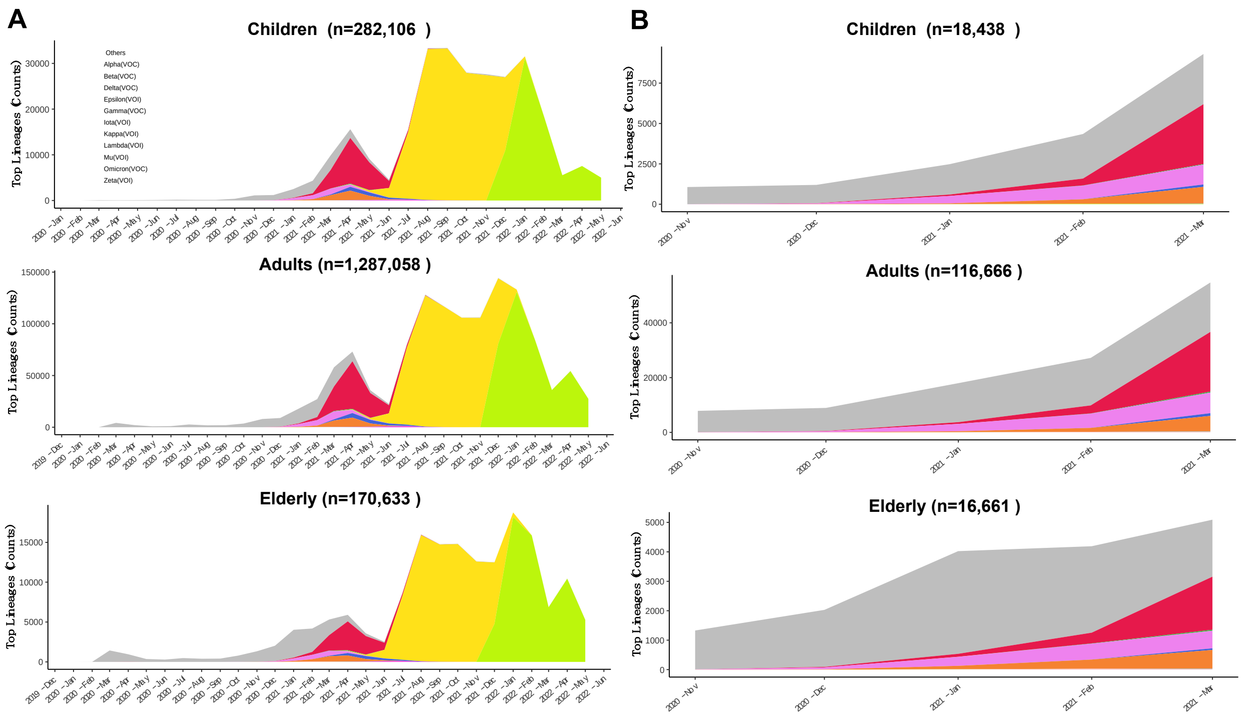

Figure S2 | Area plots reflecting the absolute abundance of variants of concern and variants of interest collected in the USA over time. (A) Absolute frequency of indicated VOCs and VOIs over time in specimens collected between January 2020 and May 2022 in the USA (n = 1,739,737 sequences, from GISAID as of May 31, 2022). (**B**) Absolute frequency of indicated VOCs and VOIs over time in specimens collected between November 2020 and March 2021. Specimens were divided into three age groups: children (up to 18 years), adults (18–64 years), and the elderly (65 years or more). The number of sequences per age group is indicated above each plot. Each subset of genomes was processed separately using the ViralVar 'Lineage Dynamics' module.

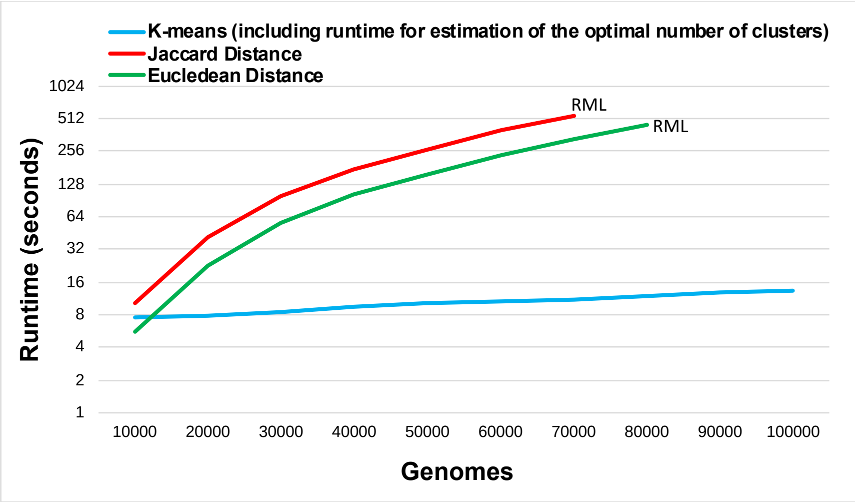

Figure S3 | Runtime performance for K-means clustering (including estimation of optimal number of clusters) and calculation of pairwise distance matrices for SARS-CoV-2 genomes (different sample size) and 86 Spike mutations. Each calculation repeated five times and average execution time reported (in seconds). All calculations were conducted in R on MacBook Pro (14-inch, 2021) with 8-core CPU, 14-core GPU, 16-core Neural Engine Accessory Kita and 32 GB memory. RML: reached memory limit.

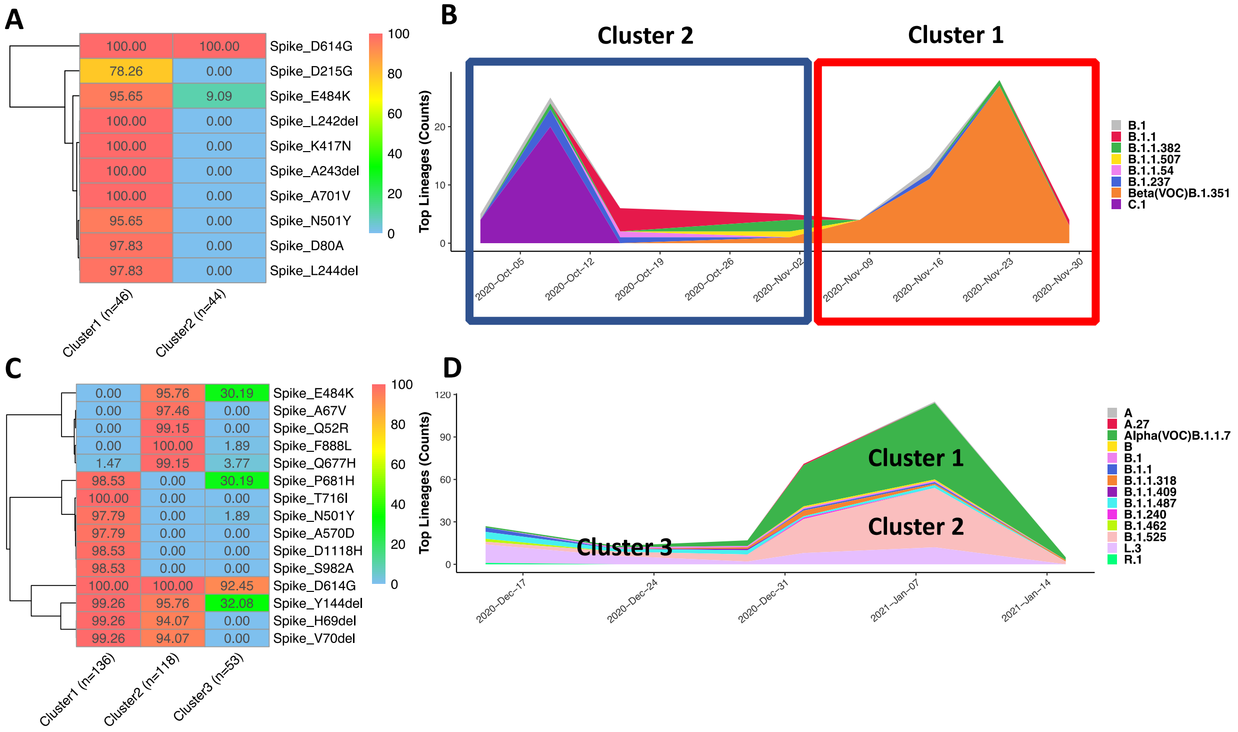

Figure S4 | ViralVar Potential in Identifying Novel Variants in Small and Local Cohorts
(A) Euclidean distance-based k-means clustering of 90 SARS-CoV-2 genomes collected in Cape Town, South Africa between October 2020 and November 2020 based on Spike mutations. **(B)** Absolute frequency of Pangolin lineages over time in 90 specimens collected in Cape Town, South Africa between October 2020 and November 2020. **(C)** Euclidean distance-based k-means clustering of 248 SARS-CoV-2 genomes collected in Nigeria between December-15, 2020 and January-15, 2021 based on Spike mutations. **(D)** Absolute frequency of Pangolin lineages over time in 248 specimens collected genomes collected in Nigeria between December-15, 2020 and January-15, 2021. Heatmaps represent the percent of genomes with a specific mutation within each cluster generated using the 'Genome Clustering' feature. Only protein mutations present in more than two thirds (70%) of genomes are shown in heatmaps. Area plots for each of Cape Town and Nigeria datasets generated by using the ViralVar 'Lineage Dynamics' module.
